# A prediction model based on deep learning and radiomics features of DWI for the assessment of microsatellite instability in endometrial cancer

**DOI:** 10.1002/cam4.70046

**Published:** 2024-08-22

**Authors:** Jing Wang, Pujiao Song, Meng Zhang, Wei Liu, Xi Zeng, Nanshan Chen, Yuxia Li, Minghua Wang

**Affiliations:** ^1^ Department of Nuclear Medicine The Affiliated Hospital of Guizhou Medical University Guiyang China; ^2^ Department of Magnetic Resonance Imaging The First Affiliated Hospital of Xinxiang Medical University Xinxiang China

**Keywords:** deep learning, diffusion‐weighted imaging, endometrial cancer, microsatellite instability, radiomics

## Abstract

**Background:**

To explore the efficacy of a prediction model based on diffusion‐weighted imaging (DWI) features extracted from deep learning (DL) and radiomics combined with clinical parameters and apparent diffusion coefficient (ADC) values to identify microsatellite instability (MSI) in endometrial cancer (EC).

**Methods:**

This study included a cohort of 116 patients with EC, who were subsequently divided into training (*n* = 81) and test (*n* = 35) sets. From DWI, conventional radiomics features and convolutional neural network‐based DL features were extracted. Random forest (RF) and logistic regression were adopted as classifiers. DL features, radiomics features, clinical variables, ADC values, and their combinations were applied to establish DL, radiomics, clinical, ADC, and combined models, respectively. The predictive performance was evaluated through the area under the receiver operating characteristic curve (AUC), total integrated discrimination index (IDI), net reclassification index (NRI), calibration curves, and decision curve analysis (DCA).

**Results:**

The optimal predictive model, based on an RF classifier, comprised four DL features, three radiomics features, two clinical variables, and an ADC value. In the training and test sets, this model exhibited AUC values of 0.989 (95% CI: 0.935–1.000) and 0.885 (95% CI: 0.731–0.967), respectively, demonstrating different degrees of improvement compared with the clinical, DL, radiomics, and ADC models (AUC‐training = 0.671, 0.873, 0.833, and 0.814, AUC‐test = 0.685, 0.783, 0.708, and 0.713, respectively). The NRI and IDI analyses revealed that the combined model resulted in improved risk reclassification of the MSI status compared to the clinical, radiomics, DL, and ADC models. The calibration curves and DCA indicated good consistency and clinical utility of this model, respectively.

**Conclusions:**

The predictive model based on DWI features extracted from DL and radiomics combined with clinical parameters and ADC values could effectively assess the MSI status in EC.

## Graphical abstract

We developed a prediction model based on diffusion‐weighted imaging features extracted from deep learning and radiomics, and combined with clinical parameters and the apparent diffusion coefficient value which could effectively assess the microsatellite instability status in endometrial cancer (EC), thereby providing a novel tool for clinicians to manage patients with EC.

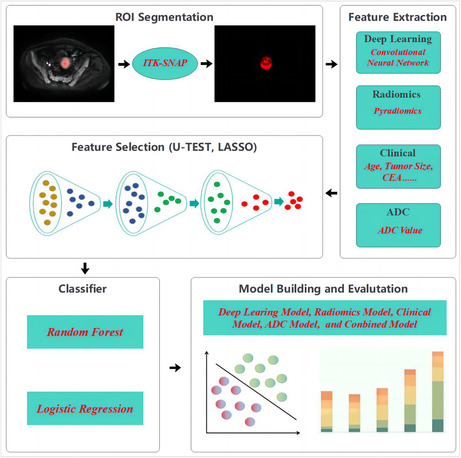

## 1 INTRODUCTION

Endometrial cancer (EC) stands as a profoundly debilitating disease affecting the female reproductive system, with roughly 30% of all patients exhibiting microsatellite instability (MSI).^1^, ^2^, ^3^ MSI status is an important prognostic biomarker for EC patients, and when patients receive immunotherapy, MSI‐H patients can achieve better treatment outcomes compared to other types of patients.^4^, ^5^, ^6^ Conventional testing assays such as immunohistochemistry (IHC), polymerase chain reaction, and next‐generation sequencing techniques, are not only labor‐intensive but also invasive and expensive for patients.^7^, ^8^ Therefore, the development of a convenient, economical, and noninvasive method to detect the MSI status of EC is important for the well‐being of affected patients.

Diffusion‐weighted imaging (DWI) is a quantitative magnetic resonance imaging (MRI) technique that has found widespread application in diagnosing and assessing EC.^9^, ^10^ It excels at not only providing detailed morphological information about lesion location and quantity at the macroscopic level but also quantifying the rate of water molecule movement within biological tissues at a microscopic scale.^11^ The evolution of information technology has paved the way for the development of computer‐aided diagnosis systems in the field of medical imaging. Radiomics represents a significant breakthrough in this domain, enabling the discovery and exploitation of higher‐order image features that might be challenging for radiologists to discern, thereby enhancing diagnostic accuracy.^12^, ^13^ Several studies have demonstrated the utility of radiomics in risk stratification,^14^ clinical classification,^15^ and prognostic prediction^16^ for patients with EC. Deep learning (DL) represents a further evolution of radiomics. Compared with conventional radiomics, DL uses multi‐level convolutional neural networks (CNN) to extract image information, resulting in highly selective and robust image features that improve the accuracy of disease diagnosis and assessment.^17^, ^18^ For instance, Chen et al. demonstrated the effectiveness of DL in assessing the depth of myometrial infiltration in patients with EC,^19^ while Tao et al. reported the capacity of DL to enhance the accuracy of EC diagnosis.^20^ Although a limited number of studies have explored the value of radiomics in assessing MSI in EC, it is noteworthy that most of these studies have relied on radiomics features from conventional MRI sequences [T1‐weighted imaging, T2‐weighted imaging (T2WI)] or computed tomography.^21^, ^22^, ^23^ Moreover, the integration of DL features has been largely absent from this research endeavor.

Therefore, this study aimed to explore the feasibility of a predictive model based on DWI features extracted from DL and radiomics combined with clinical parameters and the apparent diffusion coefficient (ADC) value in differentiating between microsatellite stability (MSS) and MSI, This endeavor aimed to introduce a novel, noninvasive pretreatment tool for evaluating the MSI status in patients with EC.

## 2 METHODS

### 2.1 Study participants

Initially, data were retrieved from 276 patients who underwent pelvic MRI for suspected EC between June 2019 and August 2023. The exclusion criteria were as follows: (i) patients with pathologically confirmed non‐EC (*n* = 25); (ii) patients without MSI status assessment (*n* = 90); (iii) patients with DWI sequences that were absent or of poor quality, making them unsuitable for analysis (*n* = 17); (iv) patients who underwent radiotherapy, chemotherapy, or surgery before the pelvic MRI examination (*n* = 13); (v) patients with incomplete clinical or histopathological information (*n* = 15). Ultimately, 116 patients with EC were included in the study. Clinical variables included age, maximum tumor diameter, and levels of carcinoembryonic antigen (CEA), carbohydrate antigen 125 (CA 125), carbohydrate antigen 199 (CA 199), and carbohydrate antigen 153 (CA 153), which were recorded at baseline. This study was approved by the local ethics committee, and the need for informed consent was waived.

### 2.2 MRI protocol

MRI scans were performed using a 1.5 T (Optima MR360, GE, Milwaukee, USA) or 3.0 T (uMR780, United Imaging, Shanghai, China; Signa HDxt/Discovery 750 W, GE, Milwaukee, USA) MRI scanner. All patients were placed in the supine position, feet first. The scanning field encompassed the area from the anterior superior iliac spine to the pubic symphysis. Axial T2WI and DWI data were obtained for subsequent analysis. Details are explained in Table 1.

**TABLE 1:** MRI acquisition parameters.

Scanner	GE Healthcare Optima MR360 1.5 T	GE Healthcare Signa HDxt 3.0 T	GE Healthcare Discovery 750 W 3.0 T	United Imaging Healthcare uMR780 3.0 T
DWI	Axial, 2D EPI	Axial, 2D EPI	Axial, 2D EPI	Axial, 2D EPI
TR/TE (ms)	2360/76.4	3327/72.4	4120/68.9	2985/75.3
Flip angle (°)	90	90	90	90
Slice/gap (mm)	5/1	5/1	5/1	6/1
Matrix	280 × 208	260 × 280	128 × 128	202 × 256
FOV (mm)	380	380	380	360
*b* (s/mm^2^)	800	800	800	800
T2WI	Axial, 2D FSE	Axial, 2D FSE	Axial, 2D FSE	Axial, 2D FSE
TR/TE (ms)	4588/80.2	3748/75.8	4378/88.6	5338/120.2
Flip angle (°)	120	112	110	110
Slice/gap (mm)	5/1	5/1	5/1	6/1
Matrix	320 × 256	382 × 479	384 × 384	388 × 422
FOV (mm)	380	380	380	360

Abbreviations: DWI, diffusion‐weighted imaging; EPI, echo‐planar imaging; FOV, feld of view; FSE, fast spin echo; T2WI, T2‐weighted imaging; TE, echo time; TR, repetition time.

### 2.3 Tumor segmentation

**FIGURE 1:**
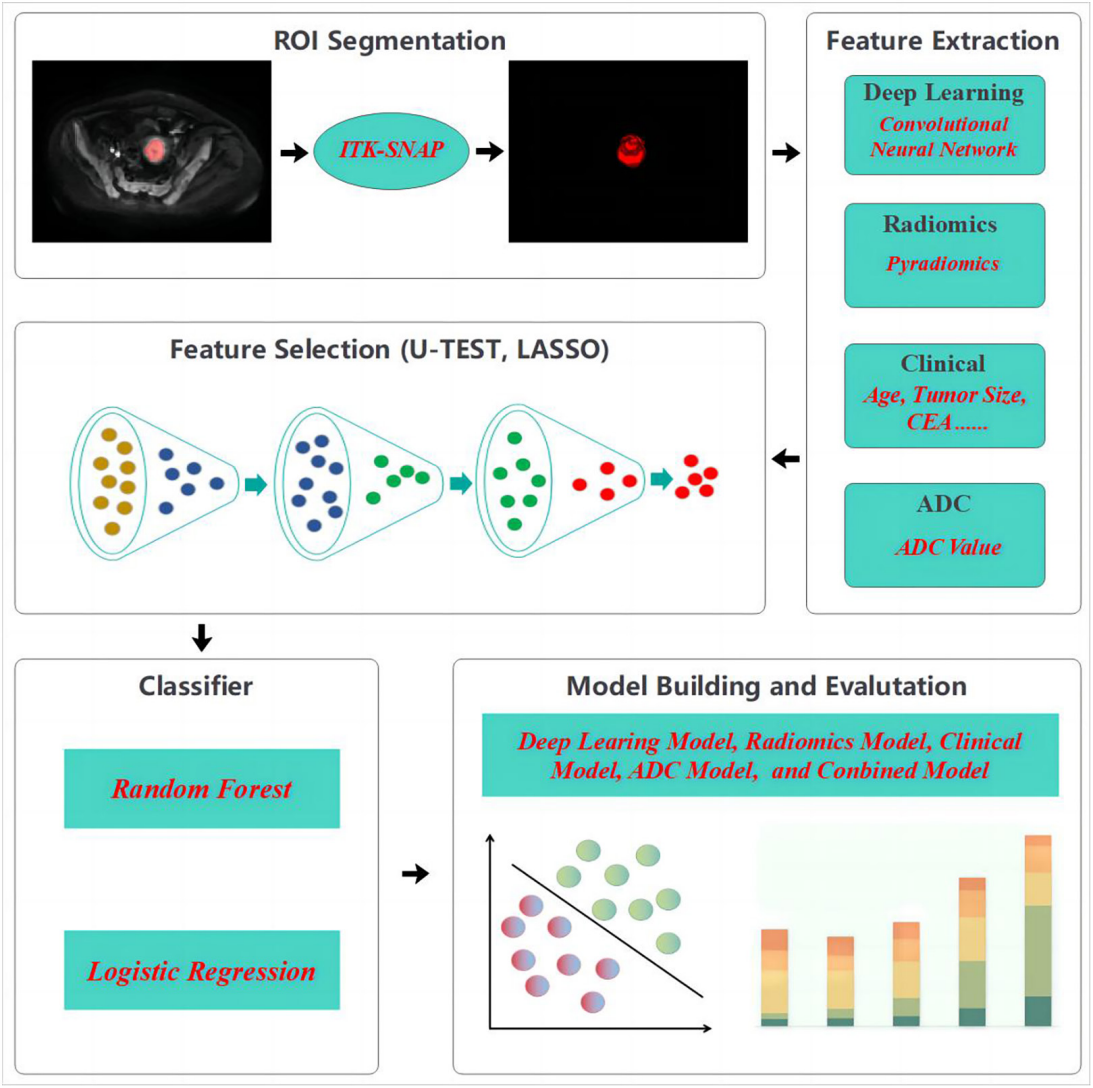
Workflow of image processing.

The ITK‐SNAP software (version 3.8.0; http://www.itksnap.org) was used for tumor segmentation. First, a radiologist with 6 years of experience (reader 1) manually delineated regions of interest (ROI) for the tumors slice‐by‐slice on the axial DWI images. Subsequently, a radiologist with 15 years of experience examined the above ROIs and determined the final results. Finally, volumes of interest (VOI) were constructed by integrating the ROIs from all the slices of each tumor using the three‐dimensional functionality of the software. The workflow of image processing is presented in Figure 1.

### 2.4 Calculation of the ADC values

The VOIs for each tumor were mapped to the corresponding ADC maps. Subsequently, the ADC values for each tumor were calculated using the following equation: *S*_*b*_/*S*_*0*_ = exp (− *b* × ADC), where “*b*” represents the diffusion sensitizing factor and “*S*_0_” and “*S*_*b*_” represent the signal intensities at a *b* value of 0 or the *b* value indicated by the subscript, respectively.

### 2.5 Radiomic features

All images were normalized using *Z*‐score normalization with a fixed bin width of 20 and voxel size resampling to 1 mm^3^ ^24^ Feature extraction was performed using PyRadiomics (version 2.1.2; https://pyradiomics.readthedocs.io), a standardized radiomics analysis workflow package.^25^ Subsequently, nine filters, including Original, BoxMean, AdditiveGaussianNoise, CurvatureFlow, BinomiaIBIurlmage, Boxsigmalmage, Normalize, Laplacian Sharpening, and DiscreteGaussian were applied. As a result, 824 radiomics features were extracted from each of the DWI images.

### 2.6 DL features

**FIGURE 2:**
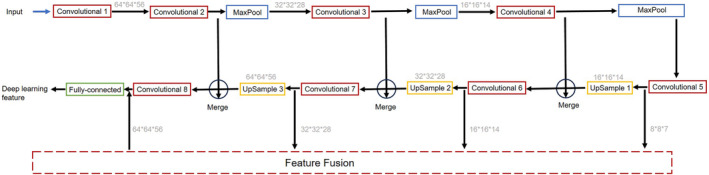
Schematic of deep learning feature generation. The model consists of eight convolutional layers with ReLU activation (red), three maximal pooling layers (blue), three up‐sampling layers (yellow), and a full sampling layer (purple).

The DWI data underwent initial preprocessing and enhancement, including conversion of Digital Imaging and Communications in Medicine files to the Neuroimaging Informatics Technology Initiative file format, alignment, resampling to achieve uniformly isotropic resolution (1 mm^3^), and random axis mirror flipping. Subsequently, a multi‐scale CNN‐based DL feature extraction model was devised to refine and extract tumor features. This model comprised eight convolutional layers with Rectified Linear Unit activation, three maximal pooling layers, three upsampling layers, and one fully sampled layer. The multi‐scale network design comprises five bottom‐up layers and three merged layers, wherein the larger scale focuses on the tumor's larger aspects while the smaller scale captures richer contextual information. During each training iteration of the model, the DWI were preprocessed separately and fed into the model. The DL features generated by the model were then obtained through the fully connected layer.^26^ The above process is shown in Figure 2. All these operations were performed using self‐written code (Supplementary 1) in Python (version 3.1.0), resulting in 128 DL features being extracted from each of the DWI.

### 2.7 Feature selection

On DWI images from 20 randomly selected patients, inter‐ and intra‐observer agreement of features was assessed. Reader 1's repeated segmentation of the VOI on DWI 2 weeks later was used to estimate intra‐observer reproducibility. To assess inter‐observer reproducibility, reader 2, another radiologist with 17 years of experience, also independently delineated VOIs and extracted features in the same manner. For radiomics and DL features, robust features were first selected by using inter‐ and intra‐observer interclass correlation coefficients >0.75 as a criterion. Then, all features were subjected to normalization using the *Z* score method. Redundant features were subsequently eliminated using the Mann–Whitney *U* test and the least absolute shrinkage and selection operator algorithm.

### 2.8 Model development

The training set was formed by randomly selecting 70% of the patients from the MSS and MSI groups, while the test set was comprised of the remaining 30% of patients. In the process of developing the model, one preprocessor (min_max_scaler) and two classifiers [logistic regression (LR) and random forest (RF)] were employed. Details of LR: penalty factor, C; category weight, none; penalty factor, I2; classification threshold, 0.5; tolerance 0.0001. Details of RF: class weight, none; maximum depth of tree, 2; criterion method, gini; minimum number of tree leaf, 1; threshold, 0.5. Ten (2 × 5) prediction models were generated, including four independent models (clinical, radiomics, DL, and ADC), and one combined model (clinical + radiomics + DL + ADC). The final prediction model had to meet specific criteria to prevent overfitting and ensure robustness: the area under the curve (AUC) values for the training and test sets should be maximized while ensuring the difference in the AUC between the two sets remained <0.15.^27^

### 2.9 MSI status assessment

The MSI status was assessed by IHC staining of four mismatch repair (MMR) proteins, MMR genes mutL homolog 1, mutS homolog 2, mutS homolog 6, and PMS1 homolog 2. EC tissues without deletion of these MMR proteins were categorized as MSS, while EC tissues with deletion of one or more of these MMR protein expression deficiencies were categorized as MSI.^28^ The above work was carried out independently by two experienced pathologists, and disagreements, if any, were resolved by negotiation.

### 2.10 Statistical analysis

Data analyses were performed using SPSS 26.0 (IBM) and RStudio 4.3.1 (R Foundation) software. Differences in variables were analyzed using the Mann–Whitney *U*‐test or the chi‐square test. The diagnostic performance of the model was quantified using the area under the receiver operating characteristic (ROC) curve. The DeLong analysis, net reclassification index (NRI), and total integrated discrimination index (IDI) were used to assess the added value of the model. The calibration curve and decision curve analysis (DCA) were employed for model validation and net clinical benefit assessment, respectively. Statistical significance was set at *p* < 0.05.

## 3 RESULTS

### 3.1 Clinical and ADC data

The training set comprised 81 patients, including 29 MSI and 52 MSS cases. The test set comprised 35 patients, including 13 MSI and 22 MSS cases. No significant differences were observed in clinical and ADC data between the training and test sets. In the training set, the CEA levels in the MSI group exceeded those in the MSS group, although, in the test set, there was no significant difference in CEA levels between the two groups. The ADC values in the MSI group were significantly lower than those in the MSS group, a trend observed consistently in the training and test sets. No significant differences were observed in age, maximum diameter, CA 125, CA 153, and CA 199 between the MSS and MSI groups, whether in the training or test set. The details are summarized in Table 2.

**TABLE 2:** Summary of characteristics in training and testing sets.

Variables	Training set (*n* = 81)			Test set (*n* = 35)			*p* value (training vs. test)
	MSI (*n* = 29)	MSS (*n* = 52)	*p* value	MSI (*n* = 13)	MSS (*n* = 22)	*p* value	
Age (year)	55.66 ± 6.15	54.52 ± 9.28	0.511^a^	58.38 ± 7.19	57.45 ± 7.84	0.724^a^	0.103^a^
Max. diameter (mm)	42.90 (13.15, 55.10)	27.45 (7.90, 43.05)	0.051^b^	29.30 (13.35, 39.95)	29.95 (8.80, 47.93)	0.827^b^	0.888^b^
ADC value	0.87 (0.74, 1.03)	1.25 (1.02, 1.38)	<0.001^b^	1.01 (0.73, 1.32)	1.27 (1.01, 1.55)	0.038^b^	0.345^b^
CA‐125 (u/mL)	23.00 (18.14, 41.06)	20.24 (14.88, 32.90)	0.120^b^	19.63 (11.67, 35.00)	20.56 (14.53, 31.72)	0.880^b^	0.564^b^
CA‐153 (u/mL)	9.02 (7.47, 10.52)	8.39 (6.79, 11.20)	0.478^b^	10.02 (8.02, 12.65)	8.55 (7.41, 10.44)	0.335^b^	0.390^b^
CA‐199 (u/mL)	16.78 (11.86, 50.40)	14.50 (10.80, 18.32)	0.102^b^	18.25 (16.05, 35.99)	14.13 (11.25, 19.54)	0.067^b^	0.385^b^
CEA (ng/mL)	1.87 (1.22, 3.02)	1.55 (1.03, 1.99)	0.025^b^	1.92 (1.57, 2.97)	1.42 (1.28, 2.24)	0.113^b^	0.622^b^

Abbreviations: ADC, apparent diffusion coefficient; CA‐125, carbohydrate antigen 125; CA‐15, carbohydrate antigen 153; CA‐19, carbohydrate antigen 199; CE, carcinoembryonic antigen; MS, microsatellite stability; MSI, microsatellite instability.

^a^ Comparisons were performed by independent *t*‐test.

^b^ Comparisons were performed by Mann–Whitney *U* test.

### 3.2 Prediction model

**FIGURE 3:**
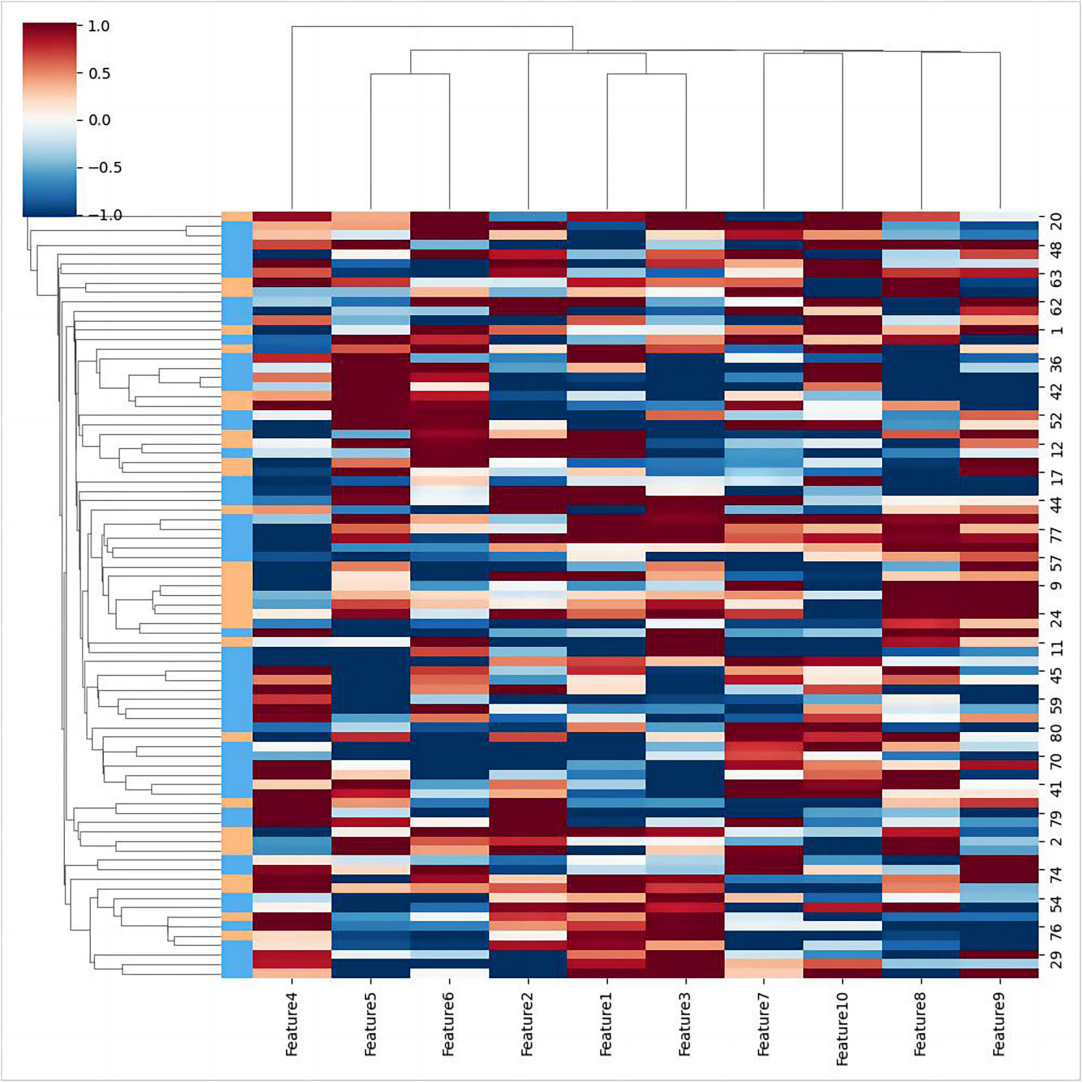
The heat map of features in combined model (clinical + DL + radiomic + ADC), features 1–11 represent additive gaussiannoise_glszm_SmallAreaHighGrayLevelEmphasis, normalize_glszm_SmallArea Emphasis, normalize_gldm_DependenceEntropy, 35th, 59th, 84th, 97th, CEA, CA199, and ADC.

The final clinical model was an LR‐based model comprising two clinical variables, CEA and CA 199. The final ADC model was an LR‐based model comprising the ADC value. The final radiomics model was an RF‐based model comprising three radiomics features, additivegaussiannoise_glszm_smallareahighgraylevelemphasis, normalize_gldm_dependenceentropy, and normalize_ glszm_smallareaemphasis. The final radiomics model was an RF‐based model comprising four DL features, namely 35th, 59th, 84th, and 97th. The final combined model (clinical + radiomics + DL + ADC) was an RF‐based model comprising the above two clinical variables, three radiomics features, four DL features, and the ADC value (Table 2 and Figure 3).

### 3.3 ROC analysis

**FIGURE 4:**
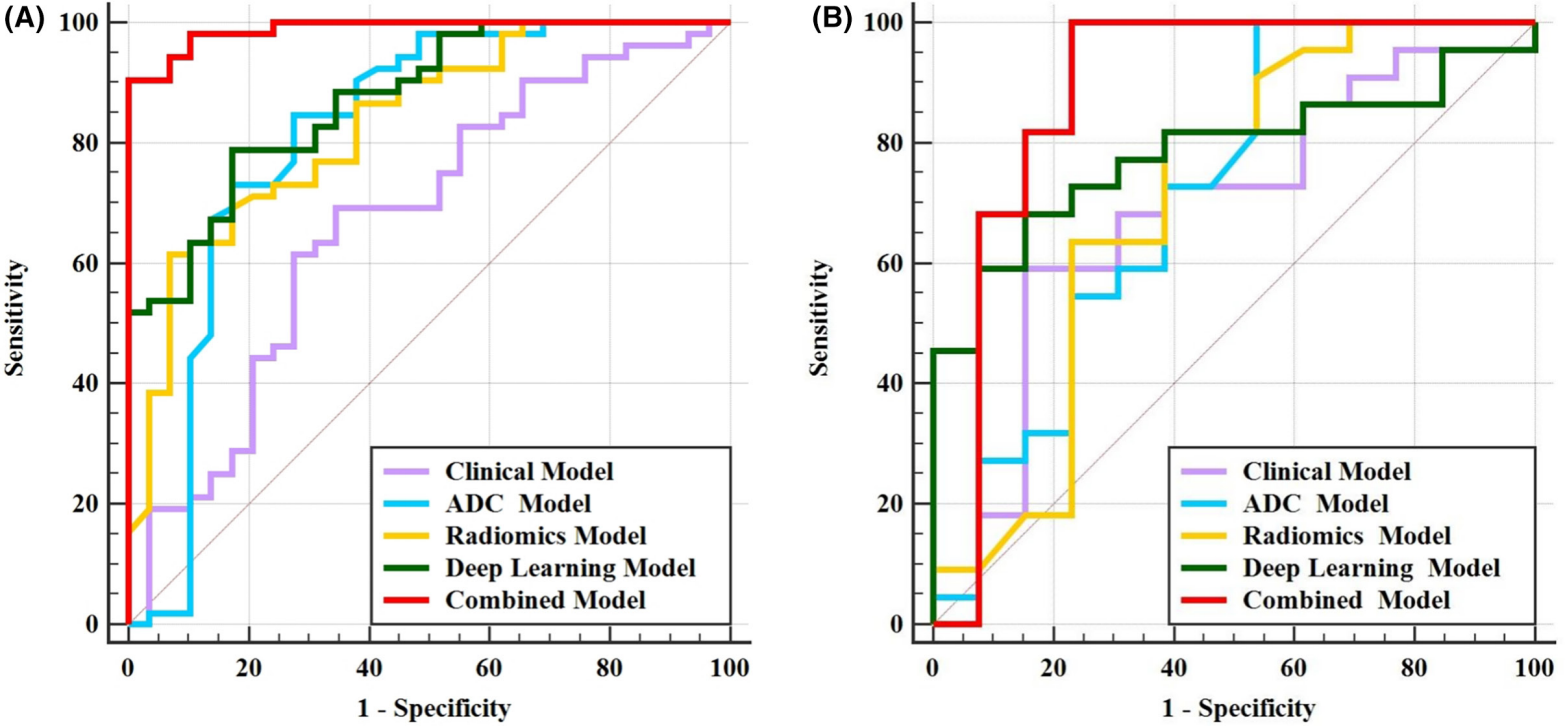
The area under the receiver operating characteristic curves of clinical (purple), DL (green), radiomic (yellow), ADC (blue), and combined (clinical + DL + radiomic + ADC, red) models for the training (A) and test (B) sets.

Compared with the clinical, radiomics, DL, and ADC models, the combined model (clinical + radiomics + DL + ADC) exhibited improved diagnostic performance in the training and test sets, with AUC values of 0.989 and 0.885, respectively. In the training set, the difference in the AUC between the combined model and the clinical (AUC = 0.671), radiomics (AUC = 0.833), DL (AUC = 0.873), and ADC (AUC = 0.814) models were all significant (*Z* = 5.043, 3.516, 2.980, and 3.061, *p* < 0.001, <0.001, = 0.003, and = 0.002, respectively). In the test set, a significant difference in AUC was observed between the combined model and the clinical model (AUC = 0.685) (*Z* = 2.278, *p* = 0.023), whereas no significant differences were observed in the AUC between the combined model and the radiomics (AUC = 0.708), DL (AUC = 0.783), and ADC (AUC = 0.713) models (*Z* = 1.424, 1.183, and 1.306, *p* = 0.154, 0.237, and 0.192, respectively, Table 3 and Figure 4).

**TABLE 3:** Predictive performance of different models.

Model	Classifier	AUC (95% CI)	Sensitivity (recall)	Specificity	Positive predictive value (precision)	Negative predictive value	False positives	False negative	Accuracy
Training set									
Clinical	RF	0.865 (0.771–0.931)	84.62%	79.31%	88.00%	74.19%	12.00%	17.29%	82.72%
	**LR**	0.671 (0.558–0.771)	69.23%	65.52%	78.26%	54.29%	21.74%	45.71%	67.90%
ADC	RF	0.917 (0.834–0.967)	82.69%	82.76%	89.58%	72.73%	10.42%	27.27%	82.72%
	**LR**	0.814 (0.712–0.892)	84.62%	72.41%	84.62%	72.41%	15.38%	27.58%	80.25%
Radiomics	**RF**	0.833 (0.733–0.906)	61.54%	93.10%	94.12%	57.45%	5.88%	42.55%	72.87%
	LR	0.655 (0.541–0.757)	78.85%	55.17%	75.93%	57.14%	24.07%	42.86%	58.02%
DL	**RF**	0.873 (0.781–0.937)	78.85%	82.76%	89.13%	66.67%	10.87%	33.33%	80.25%
	LR	0.550 (0.436–0.661)	30.77%	93.10%	88.88%	42.86%	11.12%	57.14%	53.09%
Combined	**RF**	0.989 (0.935–1.000)	90.38%	96.55%	97.92%	84.85%	2.08%	15.15%	92.59%
	LR	0.762 (0.654–0.850)	84.62%	62.07%	80.00%	69.23%	20.00%	30.77%	76.54%
Test set									
Clinical	RF	0.591 (0.412–0.754)	50.00%	76.92%	78.57%	47.62%	21.43%	52.38%	60.00%
	**LR**	0.685 (0.507–0.831)	59.09%	84.62%	86.67%	55.00%	13.13%	45.00%	68.57%
ADC	RF	0.591 (0.412–0.754)	63.64%	69.23%	77.78%	52.94%	22.22%	47.06%	65.71%
	**LR**	0.713 (0.536–0.853)	100.00%	46.15%	78.86%	100.00%	24.14%	0.00%	80.00%
Radiomics	**RF**	0.708 (0.530–0.849)	81.82%	61.54%	78.26%	66.67%	21.74%	33.33%	74.29%
	LR	0.615 (0.436–0.774)	68.18%	61.54%	75.00%	53.33%	25.00%	46.67%	65.71%
DL	**RF**	0.783 (0.612–0.904)	68.18%	84.62%	88.24%	61.11%	11.76%	38.89%	74.29%
	LR	0.699 (0.521–0.842)	81.82%	76.92%	85.71%	71.43%	14.29%	28.57%	80.00%
Combined	**RF**	0.885 (0.731–0.967)	95.45%	69.23%	84.00%	90.00%	16.00%	10.00%	85.71%
	LR	0.650 (0.471–0.803)	81.82%	53.85%	75.00%	63.64%	25.00%	36.36%	80.00%

*Note*: The bold typeface indicates the final model.

Abbreviations: ADC, apparent diffusion coefficient; CI, confidence interval; Combined diagnosis, clinical + ADC + DL + radiomics; DL, deep learning; LR, logistic regression; RF, random forest.

### 3.4 NRI and IDI analysis

The combined model (clinical + radiomics + DL + ADC) demonstrated superior risk reclassification of the MSI status compared with the clinical, radiomics, DL, and ADC models. In the training set, the NRIs were 178.51%, 156.23%, 124.80%, and 174.67% (*p* < 0.001, <0.001, = 0.002, and <0.001, respectively), and the IDIs were 117.48%, 108.39%, 50.70%, and 93.01% (all *p* < 0.001), respectively. In the test set, the NRIs were 117.48%, 108.39%, 50.70%, and 93.01% (*p* < 0.001, <0.001, <0.001, and = 0.001, respectively), and the IDIs were 46.87%, 33.16%, 29.49%, and 32.09% (*p* < 0.001, <0.001, = 0.002, and = 0.001), respectively (Table 4).

**TABLE 4:** The NIR and IDI of different models.

Model	NIR (%) (95% CI)	*p*‐value	IDI (%) (95% CI)	*p*‐value
Training set				
Clinical	178.51 (157.31–199.72)	< 0.001	76.31 (66.08–86.54)	<0.001
ADC	174.67 (152.29–197.05)	< 0.001	55.33 (47.32–63.33)	<0.001
Radiomics	156.23 (128.07–184.39)	< 0.001	48.69 (36.59–60.78)	<0.001
DL	124.80 (88.54–161.06)	0.002	38.17 (22.30–54.03)	< 0.001
Test set				
Clinical	117.48 (61.47–173.49)	< 0.001	46.87 (28.95–64.78)	<0.001
ADC	93.01 (31.81–154.20)	0.001	32.09 (12.42–51.76)	0.001
Radiomics	108.39 (50.73–166.05)	< 0.001	33.16 (16.19–50.13)	<0.001
DL	50.70 (5.57–95.83)	< 0.001	29.49 (11.01–47.96)	0.002

Abbreviations: ADC, apparent diffusion coefficient; DL, deep learning; IDI, integrated discrimination index; NIR, net reclassification index.

### 3.5 Model validation and clinical benefit

**FIGURE 5:**
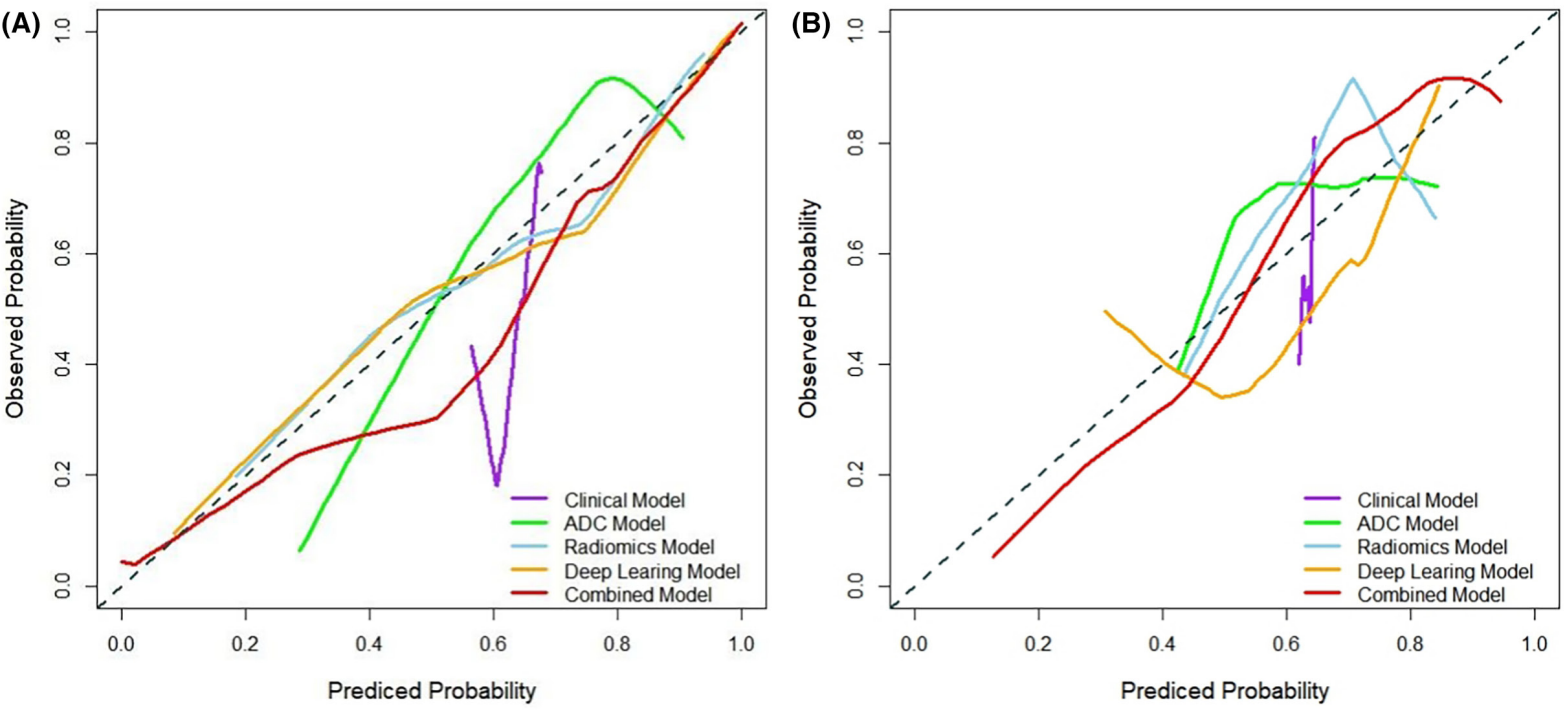
The calibration curves of the clinical (purple), DL (yellow), radiomic (blue), ADC (green), and combined (clinical + DL + radiomic + ADC, red) models for the training (A) and test (B) sets.

**FIGURE 6:**
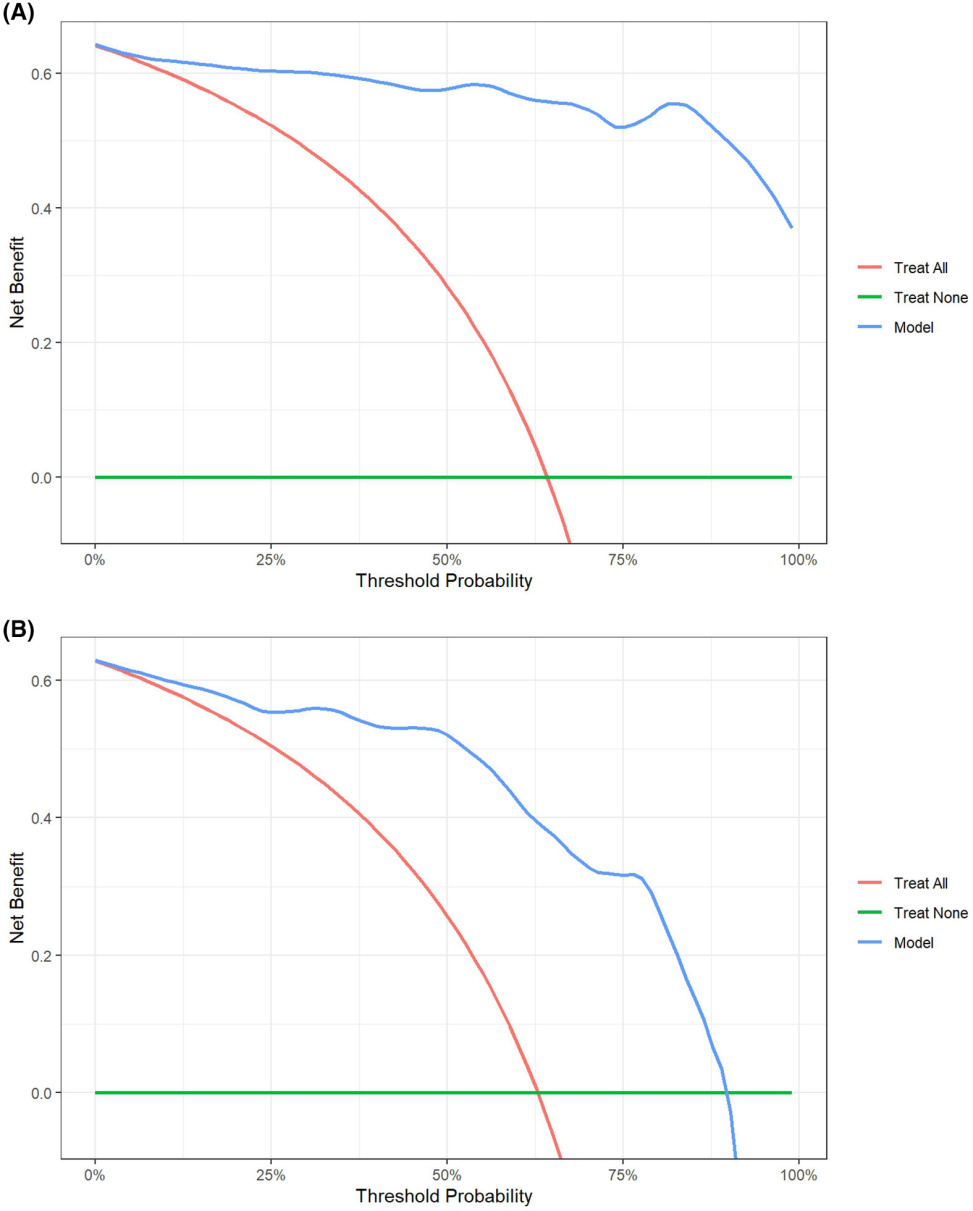
The decision curve analysis curves of the combined (clinical + DL + radiomic + ADC) model for the training (A) and test (B) sets.

In the training and test sets, the calibration curves and DCA revealed that the combined model (clinical + radiomics + DL + ADC) not only exhibited good agreement between predicted values and actual observations but also offered reliable clinical benefits for assessing the MSI status in patients with EC (Figures 5 and 6).

## 4 DISCUSSION

In this study, a combined model was constructed that integrated two clinical variables, three radiomics features, four DL features, and the ADC value to predict the MSI status in patients with EC. Furthermore, compared with the clinical, radiomic, DL, and ADC models, this combined model exhibited improved diagnostic performance in the training and test sets.

Clinical variables encompass a range of clinical indicators such as patient age, lesion size, clinical symptoms, and serum tumor markers, among others. Previous studies have shown that predictive models constructed solely using clinical variables often struggle to provide an accurate assessment of the disease state in the patients due to their limited specificity.^29^, ^30^ This study incorporated a range of variables, including age, tumor size, CEA, CA 125, CA 199, and CA 153. However, after careful consideration, only CEA and CA 199 were included in the final model, which ultimately exhibited low diagnostic efficacy. This outcome is consistent with the aforementioned studies and suggests the limited role of a clinical model in evaluating the MSI status in patients with EC. This study also revealed that CEA levels were higher in the MSI group compared with the MSS group in the training and test sets, with a significant difference observed in the training set. One possible explanation for this finding is that the MSI group tended to exhibit more malignancy, thereby resulting in higher CEA levels compared with the MSS group.^31^

ADC, a derived parameter from DWI, quantifies the rate of diffusion movement of water molecules in biological tissues.^9^ In the present study, the MSI group exhibited a higher degree of malignancy, a tighter tissue structure, and greater constraint on the water molecule diffusion movement compared with the MSS group. As a result, the ADC values in the MSI group were smaller than those in the MSS group, with diagnostic efficacies of 0.814 and 0.713 in both the training and test sets, respectively. This finding is consistent with previous studies^32^, ^33^ and further confirms the role of ADC values in assessing the MSI status in patients with EC. However, the above studies and our current investigation indicate that the diagnostic efficacy of ADC values typically ranges from 0.7 to 0.9, thereby presenting challenges in accurately determining the MSI status of patients with EC. It is speculated that this limitation might be attributed to the fact that ADC offers a single‐dimension perspective of lesion information and that there is some overlap in the range of ADC values between the MSS and MSI groups.

In this study, DL and radiomics were used to extract the features of DWI, and relevant models were constructed. Our findings underscored the effectiveness of DL and radiomics models in assessing the MSI status of patients with EC. Notably, the DL model exhibited superior diagnostic efficacy compared with the radiomics model in the training and test sets, a result in line with previous studies.^34^, ^35^ This consistency highlights the feasible and relative superiority of DL models in distinguishing MSI and MSS in patients with EC. One possible explanation for the superior performance of DL over radiomics lies in the more complex and scalable structure of CNNs. Unlike radiomics, which typically comprises a relatively fixed set of feature information, CNNs possess the capability to not only extract deeper and more subtle features but also potentially mimic the elusive subconscious process that occurs when radiologists interpret DWI.^36^, ^37^ Therefore, DL can effectively identify subtle differences between MSS and MSI, resulting in better diagnostic efficacy. Moreover, a combined model comprising clinical variables, DL features, radiomic features, and ADC values was constructed. This model demonstrated improved diagnostic efficacy and reclassification ability to varying degrees when compared with the clinical, DL, radiological, and ADC models. This finding is consistent with previous research, suggesting that a combined model integrating multidimensional validated information could provide a more comprehensive view of the lesion than a single model.^38^, ^39^ As a result, such a combined model can lead to a more accurate differentiation between MSS and MSI in patients with EC.

The choice of algorithm is a decisive factor influencing the diagnostic performance of a prediction model. In this study, the LR and RF algorithms were chosen for model construction due to their simplicity, effectiveness, and widespread use. The findings revealed that LR outperformed RF in terms of diagnostic performance for clinical and ADC models. However, for radiomics, DL, and combined models, RF emerged as the superior algorithm, a result consistent with previous studies.^27^, ^38^ The observed variations in algorithm performance could largely be attributed to differences in their characteristics. For instance, the RF algorithm is a comprehensive learning method based on bagging, affording it a greater advantage in handling classification and regression challenges.^39^ In contrast, the LR algorithm operates as a generalized linear regression analytical model, excelling in its ability to accurately predict variable effects.^40^ Additionally, it has been established that the same algorithm can exhibit significant performance differences when applied to different diseases due to the distinct pathophysiological characteristics inherent to each condition.^38^, ^39^, ^41^ Therefore, it is becoming increasingly evident that the integrated application of multiple algorithms for model development, allowing for the identification of the most suitable prediction model, might represent the future trend.

This study proposed a method for MSI status assessment in EC patients based on DWI DL and radiomics features with promising assessment performance. However, at present, pathological testing should still be preferred as the gold standard for MSI status assessment in relevant patients when conditions permit. In the future, with the continuous development of AI‐related technologies, the assessment methods proposed in this study will be further refined and may serve as a first step that has the potential to serve as an effective supplement in resource‐limited situations, thus assisting clinicians to have a more accurate evaluation of a patient's condition.

This study has certain limitations. First, the retrospective nature of the research might limit the value of the predictive model. Second, this was a single‐center study, and the cost associated with MSI testing limited the sample size. Third, the results of this study have not been externally validated, which may lead to some bias. Fourth, the DWI scanning protocols in this study were sourced from different vendors and scanners, and although standardized preprocessing was performed, it might have adversely affected the results. Finally, the study relied on two algorithms, LR and RF, for model construction, which might not have fully explored the potential for developing more stable and reliable models. In the future, attempts will be made to further expand the sample size and conduct prospective, multi‐center studies, as well as optimize the imaging protocols and incorporate additional algorithms, to achieve clinical dissemination and application of the model.

## 5 CONCLUSION

The use of a prediction model based on DWI features extracted from DL and radiomics combined with clinical parameters and the ADC value could effectively assess the MSI status in EC, thereby providing a novel tool for clinicians to manage patients with EC.

## AUTHOR CONTRIBUTIONS

**Jing Wang:** Conceptualization (lead); methodology (lead); supervision (equal); validation (equal); writing – original draft (equal); writing – review and editing (equal). **Pujiao Song:** Formal analysis (equal); funding acquisition (equal); methodology (equal); project administration (equal). **Meng Zhang:** Conceptualization (equal); data curation (equal); funding acquisition (equal); investigation (equal); resources (equal). **Wei Liu:** Formal analysis (equal); software (equal). **Xi Zeng:** Resources (equal); validation (equal). **Nanshan Chen:** Formal analysis (equal); investigation (equal). **Yuxia Li:** Funding acquisition (equal); methodology (equal); project administration (equal). **Minghua Wang:** Supervision (lead); validation (equal); writing – review and editing (equal).

## FUNDING INFORMATION

The Key Project of Henan Province Medical Science and Technology (LHGJ20220627, 2018020367), Cultivation Project of Guizhou Province for Young Talents in Science and Education (2012)173, and Youth Cultivation Fund Program of Xinxiang Medical University First Affiliated Hospital (QN‐2022‐B11).

## CONFLICT OF INTEREST STATEMENT

The authors have no conflict of interest.

## ETHICS STATEMENT

The study was approved by the Ethics Committee of The First Affiliated Hospital of Xinxiang Medical University. The authors state that they have obtained appropriate institutional review board approval or have followed the principles outlined in the Declaration of Helsinki for all human or animal experimental investigations, and the need for informed consent was waived.

## Data Availability

Data supporting the findings of this study are available within the paper and its supplementary information files.
